# Simulating the effect of different lighting conditions on left-turn driving behavior using a scenario-based anger method

**DOI:** 10.1038/s41598-022-13932-5

**Published:** 2022-06-13

**Authors:** Wu He, Jing Jing Xiong, Xuan Wang, Yan Mao

**Affiliations:** 1 College of Movie and Media, Sichuan Normal University, Chengdu, China; 2 School of Business, Sichuan Normal University, Chengdu, China

**Keywords:** Psychology, Engineering

## Abstract

Anger is a key factor affecting drivers' subjective judgment and driving skills. The influence of anger on driving behavior has been widely studied, but there is a lack of comparative research under different lighting conditions. Through a driving simulation experiment, this paper studies the influence of anger on left-turn driving behavior under two light conditions day and night. In the experiment, 32 licensed participants were divided into two groups, one in emotional neutrality and the other in anger. Among them, the emotional state of anger is induced by a traffic-related video. The results showed that compared with daytime participants, participants at night had higher anger intensity, shorter gap acceptance, and post encroachment time (PET) when left-turn driving. In addition, compared with neutral emotion participants, angry participants tended to accept shorter gap acceptance and PET when turning left. This indicates that participants failed to respond correctly to left-turn driving behavior in a state of anger. However, the response of gender differences to situational driving anger was not affected by light conditions. The anger intensity of male participants during the day and night was higher than that of female participants, and the gap between acceptance and PETs during left-turn was shorter than that of female participants. This shows that male participants are more likely to produce high-intensity anger and are more likely to make dangerous driving decisions in a state of anger. This paper puts forward some suggestions on identifying anger and preventing angry driving.

## Introduction

Traffic deaths are one of the leading causes of death worldwide. It is estimated that 1.35 million people die in road traffic accidents, and more than 5 million are injured globally each year 6. Intersections are high-risk areas with high traffic accidents^1^. Compared with about 42% in Japan, the United States, Europe, and other countries, 50% and 43%^2–4^. Left-turn driving is an important cause of traffic accidents at intersections^1^.

Left-turn driving can cause impact movements on the front or side of vehicles, increasing the risk of traffic accidents^5^. About 90% of personal injury or death is caused by human factors^6^. Driver’s anger is a key factor in traffic accidents^7^.

Anger is an emotional state that manifests itself primarily as irritability^8^. Although anger is an adaptive and functional emotion, it can also lead to many bad outcomes, such as traffic accidents caused by aggressive driving and angry driving^9^. Studies have shown that anger increases the risk of traffic and road traffic accidents^10^. Anger can hurt speeding, lane departures, traffic violations, driving performance, and risky behavior^11^. Drivers in angry states drive faster, drive closer to the vehicle in front, and accept shorter gaps when turning left^8^. Studies have shown that angry drivers exhibit more violations than neutral drivers^4^. As the human-car-road conflict intensifies, angry driving behavior is increasing^12^, and the phenomenon of angry driving "road rage" caused by anger is becoming more common^13^. Studies show that about 60.72% of motorists in China have experienced "road rage"^12^. Therefore, it is necessary to study the effect of anger on left-turn driving behavior.

Spielberger's State-The Character Anger Model^14^ is a well-known theoretical framework about the impact of anger on traffic safety. Spielberger defines the tendency to frequent and intense anger as characteristic anger, and from the temporary state of physical feelings and anger to state anger. According to this theory, Deffenbacher, Getting, and Lynch^15^ distinguish between two modes of driving anger, which are trait driving anger and situational (state) driving anger. Trait driving anger refers to the tendency of the driver to become angry while driving, while state driving anger refers to the physical arousal and anger of the driver in the event of irritable situations. Trait-based driving anger is a relatively persistent and stable personality, while situational driving anger is an emotional state. Although many studies have shown that trait anger significantly impacts driver behavior, drivers are also directly affected by situational driving anger^8,16^. Chao^17^, Ābele^10^ based on situational (state) driving anger patterns on the driver's driving performance, road decision-making, driving style, visual attention distribution, and other studies also show that situational driving anger will hurt the driver's driving behavior. However, there has been limited research on the effects of situational anger on left-turn driving behavior.

In China's traffic environment, traffic scenes such as vehicle lane change without turning lights, vehicles in intersection lanes, white solid line change, non-motorized traffic lanes, or even retrograde are very common in daily life and can arouse the anger of most drivers to varying degrees^18^. Sullman^19^ is based on the Driving Anger Scale^15^ defines four types of driving-related anger-inducing situations: impaired progress, dangerous driving, hostile gestures, and impolite driving. Wickem^20^ also points out that behaviors such as walking back and forth (33%) and driving slowly (20%) tend to cause anger among drivers. Anger is an important cause of drivers' angry driving^7^. Most traffic accidents caused by angry driving behavior occur at night^21^. However, there has never been an investigation into whether lighting conditions can cause changes in drivers’ anger.

In addition, the night driving environment is also a key factor affecting driving behavior. Night driving is dangerous for all road users^22^. Although only 21–23% of vehicle mileage^23^ is required to travel at night, 51% of fatal accidents and 29.5% of accidental injuries occur at night. Road users have three times the mortality rate at night^1^. Wood^24^ points out that dim lighting conditions can lead to a significant reduction in the driver's reaction time and pedestrian identification distance, thereby increasing the risk of driving at night. Second, driver drowsiness at night is also a major cause. Influenced by circadian rhythms, people will become sleepier at night, and drowsiness leads to reduced drivers' awakening and functional sensory-motor skills, leading to poor driving decisions^25^ However, there is little research on the effect of lighting conditions on left-turn driving behavior.

Although there has been more research on driver anger, there is a gap in the effect of anger on driver left-turn behavior. Furthermore, different lighting scenarios can influence driver behavior. Therefore, investigating the left-turn behavior of drivers with different emotions when faced with different lighting scenarios could provide vital information to control the harmful effects of situational driving anger and thus make practical recommendations for improvement. Moreover, the study is equally relevant for addressing left-turn collisions during daytime and nighttime. Therefore, the feasibility of this study is promising.

In the study of situational driving anger, the previous methods of inducing anger mainly included experience recall, story situation, and film induction^26,27^. In recent years, the film-induced method has been widely used in the study of situational driving anger, Zhang Tingru^8^, Ho Dongchao^17^, and LīvaĀbele^10^ also demonstrate the effectiveness of film-induced methods in the induction of anger.

In this paper, the driving simulator experiments under different light conditions were carried out using the film induction method to induce the participants' anger, to solve the following problems: first, will the anger be affected by the light conditions? Second, is the left turn driving behavior of participants affected by light conditions under the state of anger? The results of this study contribute to a deeper understanding of the mechanism and consequences of situational driving anger and put forward some suggestions to control the negative effects of situational driving anger.

The first section focuses on the experimental equipment, scenarios, materials, and experimental design. The second section focuses on the testing procedures involved. The third section focuses on the analysis of the data. Section IV discusses. Section V limitations and future perspectives. Section six concludes.

## Methods

### Participants

In this research, 32 participants with normal vision or correction, no color blindness or other eye diseases, and who had a driver's license and at least one year of driving experience were interviewed anonymously and voluntarily. They were randomly divided into 2 groups of 16 people, equally male to female. All the participants were persuaded that their information will remain anonymous, and the total data will be applied to research, increasing the traffic safety knowledge. Each participant is paid CNY 50. The participants were informed that they could quit the experiment at any time and were asked to sign an informed consent form before the experiment began. The study was approved by the Ethics Committee of Sichuan Normal University and all experimental procedures were performed in accordance with the Declaration of Helsinki.

### Experimental equipment

The current study is carried out using a fixed base all-instrument open cab simulator system. The driving simulator cab uses three 42-inch LED screens to provide a 180-degree horizontal view, one screen for the front view image, and two screens for the side mirror display. The simulator includes all automotive controls, such as a 1060-degree rotating feedback-powered steering wheel, clutch, brake and accelerator pedals, shifter (for manual transmissions), mirrors and side mirrors, steering signal lights, like actual cars. The simulator is equipped with a screen panel that displays the speedometer, tachometer, steering signal indicator, and the audio system in the simulator is connected to the analog scene, providing traffic sound and engine noise to reproduce the actual vehicle's surroundings, thereby enhancing the authenticity of the driving experience on the simulator.

### Experimental design

This study was designed using a 2 (control/anger group) × 2 (day/night) hybrid experiment design. The internal factor of the participant is the emotional state. In the experiment, participants' anger was induced using a 5-min film clip related to traffic filmed from the driver's perspective. Before the experiment, participants were shown a 5-min-long daytime and night video clip taken from the driver's point of view to induce anger. Participants were asked to imagine themselves driving in the video clip vehicle. Half of the participants performed left-turn tasks in anger-driven situations, while the other half completed the same tasks without anger-induced neutral emotions. The factor between the participants is the lighting conditions of the driving. Each group of participants was required to complete the left-turn driving task under different lighting conditions.

### Experimental materials

The first video is in the daytime: The participant's vehicle is driving normally in the inner lane of a four-lane city road. After about 30 s, a car overtakes the participant's vehicle in the outside lane and cuts in without using a turn signal. Participants honked their horns to warn the driver that such behavior was dangerous. But the vehicle in front began to deliberately block the participants' progress at a very slow pace. When the participant tries to get rid of the vehicle in front by changing the lane, the vehicle in front suddenly accelerates. This continued for about 3 min, with the driver of the vehicle in front throwing debris out of the window and accelerating away.

The second video is at night: The participant's vehicle is driving normally in the inner lane of a four-lane city road. After about 30 s, the vehicle in front suddenly changes lanes without turning the lights, and the participants honk their horns to indicate that the driver is dangerous. But the vehicle in front started deliberately blocking the participants' progress at a very slow speed. When the participant tries to get rid of the vehicle in front by changing the lane, the strong high beam of the vehicle coming in the opposite direction affects the participant's sight. This continued for about 3 min, with the driver of the vehicle in front throwing debris out of the window and accelerating away.

In the experiment, the difference between the two videos was that the night participants were not only affected by the rude behavior of the vehicle in front but also by the vehicle coming in the opposite direction, while the participants were mainly affected by the rude behavior of the vehicle in front. Yu^12^, Tingru^8^, Feng^18^, Chang^28^, Ren^13^. The above driving behavior will arouse the driver's anger.

### Experimental scenarios

The experiment used VR technology to virtualize an urban road approximately 8 km long and 3.5 m wide with three lanes. According to the speed limits for intersections in the traffic regulations, the speed of both through traffic and left-turning traffic during the experiment was 30 km/h. At the same time, different starting points were designed for through traffic and left-turning traffic to create traffic conflicts. The test area of the experiment were points A (30 m) and B (64 m), which farfrom the conflict area. In the experiment, the left-hander drove into the test area at a speed of 30 km/h. To avoid the perceived effect of speed changes on participants, the through train also needs to pass through the intersection at 30 km/h. In addition, participants can see that the pass-through vehicle is approaching. In this study, the conflict area is defined as the overlapping area between the left vehicle track and the straight vehicle track.

**Figure 1:**
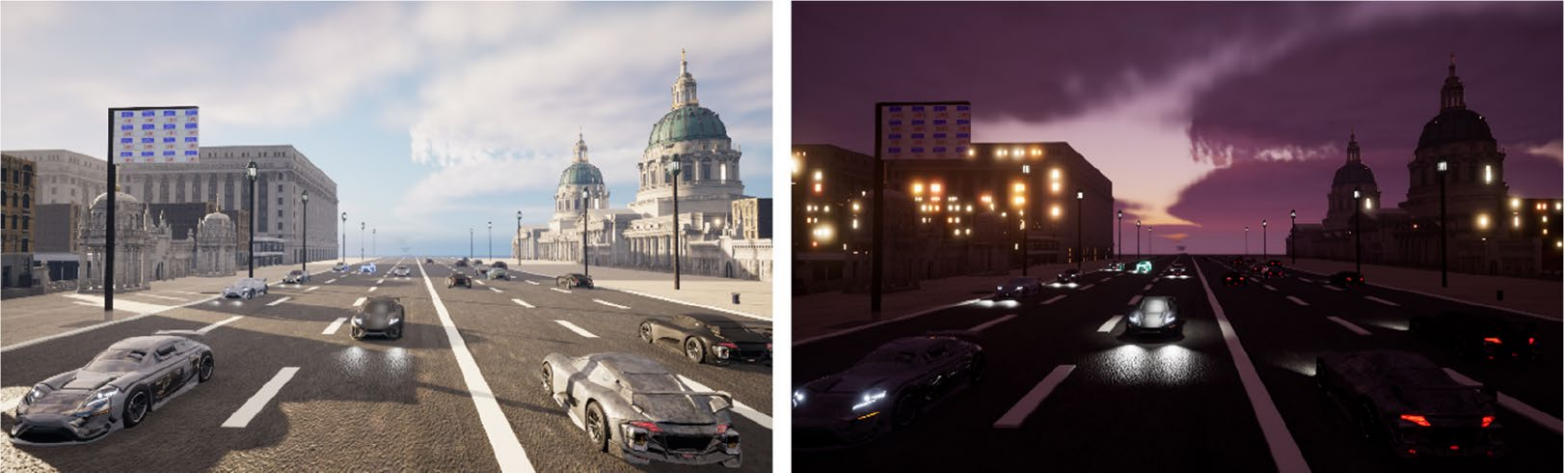
Experimental scenario diagram.

Figure 1 experimental scenario diagram shows the experimental virtual scenario with the daytime scenario on the left and the night scenario on the right.

## Procedure

Participants arrive at the lab to sign an informed consent form for the experiment and fill in the relevant personal information (age, gender, driving experience) anonymously. The experimenters then divided participants into two groups on average, and everyone has 15 min to practice driving to familiarize themselves with the simulator's operation and simulation environment. The variables involved in our experiment are summarized in Table 1.

**Table 1:** Description of the experimental variable.

Variables	Variable description	MAD (SD)		MAD (SD)	
		Calm	Anger	Gap	PET
**Experiment phase**					
Pre-induction	Participants were neutral and did not watch the video clip	5.34 (0.94)	3.25 (1.14)	–	–
Post-induction	Participants had watched the video clips and become anger	2.53 (1.14)	6.06 (0.92)	–	–
Post-drive	Participants completed the left-turn driving experiment	3.19 (1.38)	4.09 (0.82)	–	–
**Experiment conditions**					
Day	Left-turn driving in daytime	4.21 (1.53)	3.96 (1.56)	6.28 (1.25)	4.47 (1.10)
Night	Left-turn driving at night	3.17 (1.66)	4.98 (1.31)	4.75 (1.05)	3.38 (1.13)
**Traffic operation variables**					
Gap acceptance	The time interval between the opposite vehicles(s)	–	–	–	–
Post encroachment time (PET)	The time interval when the first car leaves the cross conflict point and the second car enters the conflict point(s)	–	–	–	–
**Demographic variables**					
Female	Female participants perform driving tasks	4.25 (1.67)	4.15 (1.46)	6.06 (1.34)	4.53 (1.14)
Male	Male participants perform driving tasks	3.13 (1.48)	4.79 (1.53)	4.97 (1.12)	3.31 (1.03)

Before the start of the experiment, a group of participants needed to complete the emotional assessment of the intensity of the three emotions (calm, anger, and fear) that they felt at the time, with a score of 7 (1 was not at all and 7 was very strong). Participants' mood scores were recorded and administered during the experiment. Among them, fear was not scored and managed in this experiment, because fear was not involved in this experiment.

At the beginning of the experiment, the experimenters showed one group of participants a video clip of the daytime situation, re-evaluated the participants' emotional state after watching, and then conducted a simulated driving experiment. When participants completed daytime driving tasks, they needed a 5-min break to complete the emotional score after the first experiment and calm their emotions.

**Figure 2:**
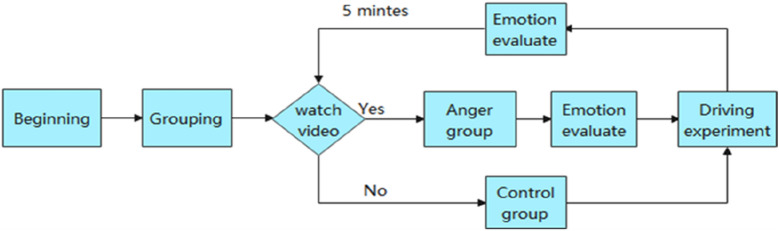
Experimental procedure.

The second experiment began. The experimenters were asked to focus again on video clips from the night-time situation and repeat the emotional score, followed by a night-time simulated driving experiment. The third emotional score was performed after the end of the experiment. The other group was an experimental control group that conducted simulated driving experiments without watching videos and mood scores. In the experiment, each group of participants was required to complete two consecutive driving sessions, one in the daytime and one at night. The flow of the experiment is shown in Fig. 2.

## Data analysis

### Situational driving anger effectiveness analysis

**Figure 3:**
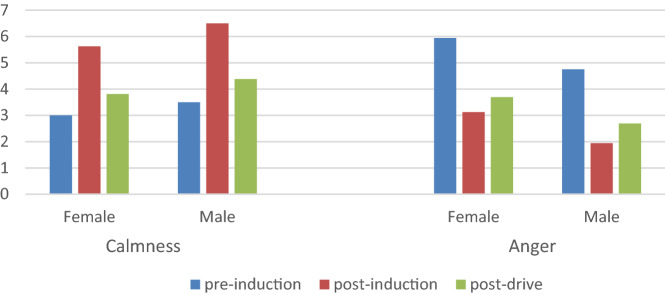
The changes of emotion intensity in different experiment phase.

The effectiveness of emotional induction affects the effectiveness of experimental results. In this study, the intensity of the three emotions (out of 7) was assessed (on a scale of 7, 1 not at all, and 7 very strong) using Jeon^11^ experimental method for participants before, after, and after the completion of the experiment, and relevant data was collected. The emotion evaluation data from 16 participants was analyzed using a 3 (experiment phases) × 2 (lighting conditions) mixed ANOVA for each participant. It can be seen in this Table 1 that compared to pre-experiment and post-experiment, the emotional intensity of participants during the experiment had changed significantly. It was noticeable that although participants had completed the experiment, participants anger intensity was still higher than when they had not watched the video, suggesting that aroused anger lasts for some time, as shown in Fig. 3.

**Figure 4:**
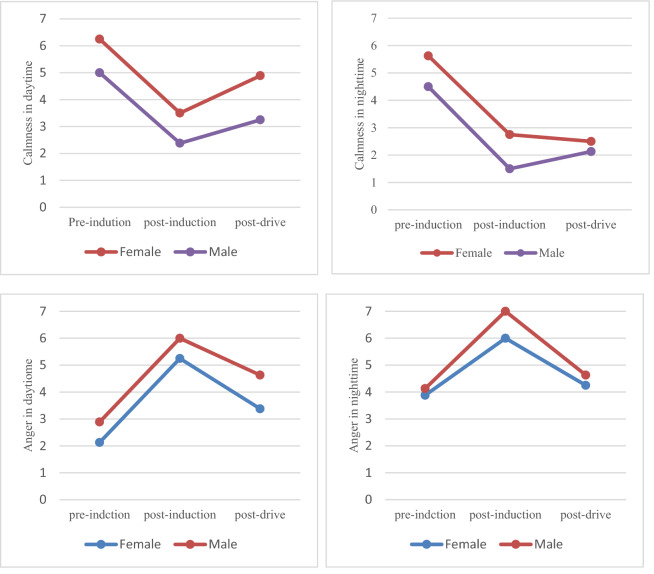
Changes in participants' emotional intensity.

Our experiment focused on the intensity of participants' anger. The experiment was subjected to a mixed ANOVA on mood between participants and a 2 (emotional state: angry/calm) × 2 (experimental phase: pre-induction/post-induction). In terms of the scores of calm emotion state, there were significant differences in emotional intensity between participants during different experiment stages (F(2,93) = 51.11, p = 1.06E − 15, Fcrit = 3.09), participants had lower levels of calm emotional intensity (5.34 vs. 2.53) after watching the video compared to the emotional intensity before the experiment; there were also significant differences in anger intensity (F(2,93) = 71.58, p = 1.51E − 19, Fcrit = 3.09), participants had a higher intensity of anger (3.25 vs. 6.06) after watching the video compared to the pre-experiment emotional intensity. These results showed that the intensity of anger changed significantly after the participants began the experiment, and it proved the effectiveness of the film-induced method used in this paper in the study of situational driving anger. As shown in Fig. 4.

### Analysis of participants' emotional intensity

**Figure 5:**
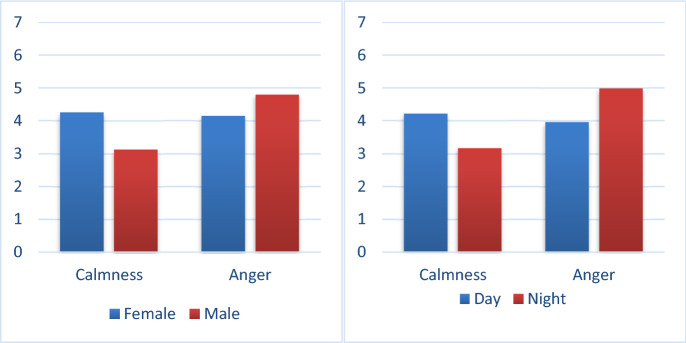
The effect of light and gender on emotional intensity.

First, we analyzed the mixed variance of the emotional scores between the participants on gender and emotions 2 (emotion state: anger/calm) × 2 (gender: male/female). In terms of calm intensity, the gender difference had a significant effect on the participants' emotional intensity (F(2,93) = 12.19, p = 0 0.0007, Fcrit = 3.94). Female participants had a higher level of calm than male participants (4.25 points vs. 3.13 points); in terms of anger intensity, the gender difference also had a significant effect on participants' emotional intensity (F(2,93) = 4.48, p = 0.04, Fcrit = 3.94). Male participants had a higher intensity of anger than female participants (4.79 points vs. 4.15 points). These results showed that male participants were more likely than female participants to develop anger and have a higher intensity of anger (as shown in Fig. 5). At the same time, we also performed an ANOVA of light and emotion 2 (emotion state: anger/calm) × 2 (lighting conditions: day/night). In terms of calm intensity, the lighting conditions had a significant effect on the participants' emotional intensity (F(2,93) = 10.26, p = 0.002, Fcrit = 3.94), participants in the daytime had a higher level of calm intensity (4.21 points vs. 3.17 points) compared to participants in the night, of anger (4.98 points vs. 3.96 points) than participants in the daytime. These results showed that participants were more likely to develop anger and have a higher intensity of anger at night. As shown in Fig. 5.

Finally, a mixed ANOVA between light and gender 2 (lighting conditions: day/night) × 2 (gender: male/female) showed that there was no interaction between light and gender on the intensity of calm (F(2,93) = 0.59, p = 0.44, Fcrit = 3.94); but a significant interaction between light and gender on anger intensity (F(2,93) = 6.01, p = 0.02, Fcrit = 3.94). As shown in Table 2.

**Table 2:** Analysis of the variance between light, gender, and emotional intensity.

Variables	Anger			Clam-ness		
	F	P-value	F-crit	F	P-value	F-crit
Light	12.06	0.0008	3.94	10.26	0.002	3.94
Gender	4.48	0.04	3.94	12.19	0.0007	3.94
Light × gender	6.01	0.02	3.94	0.59	0.44	3.94

P < a = 0.05.

**Figure 6:**
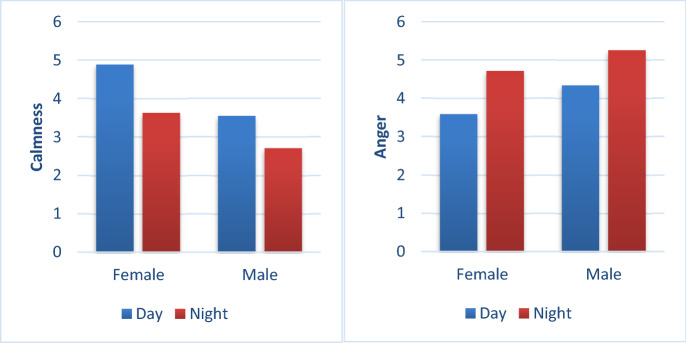
Effects of different lighting conditions on emotion.

Regardless of lighting conditions, male participants had a higher intensity of anger than female participants (4.33 points vs. 3.58 points; 5.25 points vs. 4.71 points). These results showed that male participants were more likely than female participants to have a higher intensity of anger. As shown in Fig. 6.

### Analysis of the effect of situational driving anger on left-turn behavior

**Figure 7:**
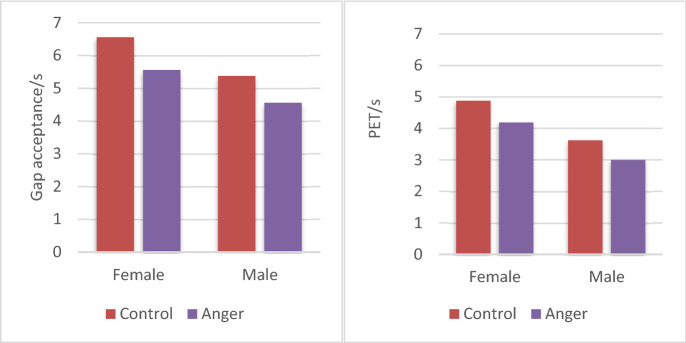
The influence of different emotional states on left-turn behavior.

**Figure 8:**
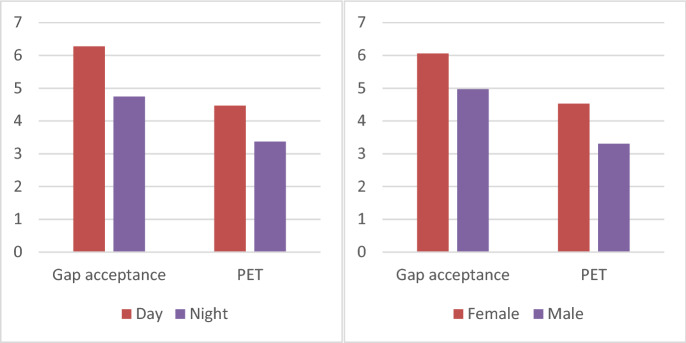
The effect of light and sex on left-turn behavior.

The driving data from the 32 participants was analyzed using a 2 (emotional state) × 2 (lighting conditions) mixed ANOVA for each traffic experiment. As shown in Table 3. First, we did a variance analysis of gap acceptance and post encroachment time (PET) between the subjects on the left-turn behavior 2 (emotion state: anger/control). The results showed that in terms of the gap acceptance of the left-turn task, there are significant post encroachment time (PET) can’t difference in emotion (F(1,62) = 8.08, p = 0.006, Fcrit = 3.99). In terms of the post encroachment time (PET), differences in emotion can also cause significant differences (F(1,62) = 4.76, p = 0.032, Fcrit = 3.99). These results showed that participants who were in situational driving anger had shorter gap acceptance (5.06 s vs. 5.97 s) in left-turn tasks and shorter post encroachment time (PET) (3 59 s vs. 4.25 s) (as shown in Fig. 7). Secondly, the results show that the difference in lighting conditions also has a significant effect on the left-turn behavior of the participants. From the gap acceptance of the left-turn task, there are significant differences in the different lighting conditions (F(1,62) = 30.42, p = 7.21E − 07, Fcrit = 3.99). In terms of the post encroachment time (PET) of the left-turn task, there are also significant differences in lighting conditions (F(1,62) = 15.32, p = 0.0002, Fcrit = 3.99). These results showed that participants at night had shorter gap acceptance (4.75 s vs. 6.28 s) and shorter post encroachment time (PET) (3.38 s vs. 4.47 s) when turning left than participants in the daytime (as shown in Fig. 8).

**Table 3:** Participants' left-turn behavior variance analysis results.

Variables	Gap acceptance		PET
**Emotion state**			
Control	Mean	5.97	4.25
	SD	1.28	1.19
Anger	Mean	5.06	3.59
	SD	1.27	1.21
ANOVA	F	8.08	4.76
	P-value	0.0006	0.032
	Fcrit	3.99	3.99
**Lighting condition**			
Day	Mean	6.28	4.47
	SD	1.25	1.10
Night	Mean	4.75	3.38
	SD	1.05	1.13
ANOVA	F	30.42	15.32
	P-value	7.21E − 07	0.0002
	Fcrit	3.99	3.99
**Emotion** × **light**			
Mixed ANOVA	F	0.13	0.66
	P-value	0.71	0.42
	Fcrit	4.001	4.001

P < 0.05.

**Figure 9:**
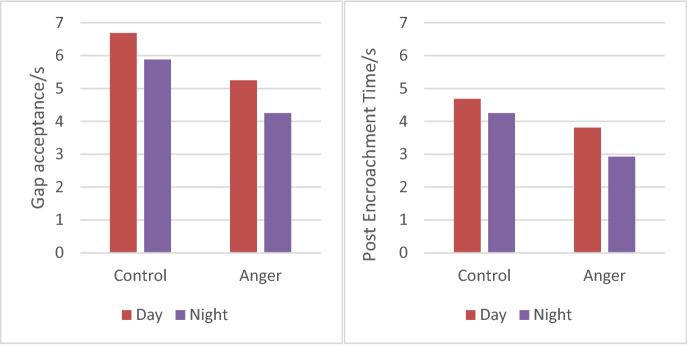
The effect of different lighting conditions on left-turn behavior.

The experiment also tested 2 (emotional state: anger/control) × 2 (illumination condition: day/night) × 2 (behavior: time after gap acceptance/encroachment) to investigate whether different emotional and illumination conditions had an effect on left-turn behavior. The results showed that there was no interaction between different emotions and light conditions on gap acceptance of the participants' left turn behavior (p = 0.72 > a = 0.05); there was also no interaction between the different emotions and light conditions on the post encroachment time (PET) of the participants' left turn behavior (p = 0.42 > a = 0.05). These results indicated that participants showed no significant differences in left-turn driving behavior during daytime and nighttime anger (as shown in Fig. 9).

Finally, to test for gender differences in the experiment, a mixed ANOVA of four variables2 (emotion: anger/control) × 2 (lighting condition: day/night) × 2 (behavior: gap acceptance/time after encroachment) × 2 (gender: male/female) was conducted. The results of the experiment showed that gender differences were not significant.

## Discussion

### Analysis of the effectiveness of the film-induced method

The focus of this study was the intensity of anger among participants before and after the start of the experiment. In the experiment, the change in mood after watching the video clip was derived, i.e., a decrease in the intensity of calm mood (5.34 points vs. 2.53 points) and an increase in the intensity of anger (6.06 points vs. 3.25 points). The results indicate that the participants' anger was successfully aroused. This is consistent with the study of Zhang^8^, that is, the film-induced method is effective in situational driving anger research. Compared to other interaction methods, the experimental method based on the driving simulator may be considered to lack ecological effectiveness. Our experiment is very similar to the experience of anger while driving. In actual traffic, although an event that provokes anger usually lasts only a few minutes, the anger it had aroused may continue into a subsequent driving situation and has an impact on driving behavior^29^. Some people may think that the anger may quickly disappear when performing demanding driving tasks. However, the results showed that despite the decrease in anger intensity during the driving simulation experiment (4.09 vs. 6.06 points), however, the anger is still significantly higher than before the anger induction (4.09 points vs. 3.25 points).

### Effect of lighting conditions on situational driving anger

In conclusion, the results of this study show that the anger intensity of participants at night is significantly higher than that during the day. Because the risk of driving at night is higher than during the day, and the reduced visibility at night increases participants’ cognitive burden on the environment^30^, participants are more likely to have a high degree of anger. Relevant studies show that drivers will not reduce their speed due to reduced visibility at night^31^. Therefore, participants need to focus on the surrounding environment to ensure the safety of driving behavior. When this attention is interrupted by sudden rude behavior, participants will instinctively produce stress psychological response, resulting in the increase in angry emotional intensity^13^. At the same time, due to the influence of circadian rhythm, humans usually become sleepier at night^32^. Driving for a long time makes the participants bear both physical and mental pressure. Participants need to maintain a high degree of tension to deal with various possible situations. When the front party suddenly rude driving behavior, participants will be more sensitive to rude provocation, resulting in an increase in anger. This is consistent with Li Ming's^21^ research, that is, before the outbreak of "road rage", the actor's psychology mostly had adverse tendencies and pathological signs such as anxiety, tension, irritability, depression, impetuosity, anger, evasion, and jealousy. Yu^12^ also pointed out that the more frequent you drive, the more mileage you drive, the easier it is to drive angrily.

The results showed that male participants were angrier than female participants regardless of light conditions. The main reasons for this difference between male and female subjects are as follows: First, women are more likely than men to predict the potential risks and future consequences of their behavior. They will avoid any violent behavior that may cause each other's anger to form a self-protection mechanism^33^. Secondly, female drivers respect the rules more than male drivers. Meanwhile, compared with female drivers, male drivers have relatively low public self-awareness, which leads male participants to pay more attention to their emotions and have a higher degree of anger when encountering abnormal driving behavior^34^. Finally, compared with male participants, female participants were more likely to adopt a more adaptive attitude to express anger, while male participants were more aggressive. The research of Ming^21^, Yan^35^ and others shows that male participants have a higher probability of anger during driving than female participants. But Bjorklund^36^, Cong Haozhe^37^, LīvaĀBele^10^ and others pointed out that female drivers are more likely to produce road anger. Pelin Deniz^9^ also showed that masculinity and femininity regulate the relationship between driving anger and driving anger expressions of young drivers. The impact of gender differences on anger is controversial. In this study, our experimental results show that gender differences have a significant impact on the degree of the anger of participants.

### The effect of situational driving anger on left-turn behavior

The results of this paper showed that participants who were in a situational driving rage had shorter gap acceptance and post encroachment time (PET) when turning left than participants with neutral emotions. This is consistent with studies by Zhang Tingru^8^, in which participants in anger had shorter wait times when turning left, resulting in shorter acceptable gaps. Beatriz González-Iglesias^6^ points out that anger can lead to traffic violations by drivers. Li et al.^38^. It is also confirmed that angry drivers tend to show a higher risk-taking tendency. From a behavioral perspective, driving anger can adversely affect driving behavior, increasing the risk of traffic accidents. Secondly, visual perception is the main source of driving information. Easterbrook^39^ pointed out that high awakening negative emotions (such as anger) can lead to tunnel vision, a peripheral vision loss, but the central vision remains the same. Therefore, the horizons of drivers in situational driving anger will be narrow, and participants will focus mainly on traffic events ahead and miss safety–critical information in the surrounding view. All of these factories lead to wrong driving behavior decisions. Zhang^8^ also pointed out that situational driving anger reduces the range of the driver's visual attention. Taken together, anger leads to incorrect driving decisions made by participants that increase the risk of traffic accidents.

### Effect of lighting conditions on left-turn behavior

The experimental results showed that the gap acceptance and post encroachment time (PET) was shorter when the participants performed left-turn driving at night (6.06 s vs. 5.5 s; 4.06 s vs. 4.53 s). The main reasons are as follows: First, visual perception is the main source of driving information. The limited exposure range of vehicle headlights reduces participants' visibility on the road at night, resulting in reduced awareness of other vehicles on the road and the surrounding environment, resulting in incorrect decisions. Second, participants' drowsiness at night was also a major cause. Horne's^32^; Lowden^25^ research shows that drowsiness can lead to reduced wake-up and functional sensory-motor skills, leading to more dangerous driving behavior. Influenced by circadian rhythms, participants become sleepier at night, leading to poor driving decisions. At the same time, the lucky mentality and the safe enclosed interior space also make participants more likely to take a chance at night. Research by Li^21^ also points out that accidents caused by road rage occur mostly at night. Finally, the intersection is a complex part of the road network, where participants need to make multiple decisions at considerable temporal pressure and high speed^40^, while the dim environment at night increases the cognitive burden of participants, causing participants to be unable to make correct decisions promptly. In conclusion, participants had a shorter gap in acceptance and post encroachment time (PET) when turning left at night.

## Limitation and future perspectives

This study has some limitations, but they could be addressed in the future. First, the driver's reaction in the simulator differs from the real world, and the adopted simulator adopted reduces the authenticity and effectiveness of the experimental results. In the future, the comprehensive use of modern technologies such as VR and EEG can be used to reduce the authenticity of the experimental results and the real-world difference.

Second, the participants' emotional scores were mainly self-reported data, and it was not possible to determine whether the participants had the mentality to meet social expectations. The study could consider objective data collection methods to minimize data errors. Third, other situations besides state driving anger that could cause participants' anger was also not considered, and the impact of verbal aggression on driver driving anger is an interesting topic for future research. Finally, most of the study samples are young drivers with small sample size, and the experimental results are not generally representative. In the future, the study sample size should be considered to expand, and more participants with different driving experiences should be selected for experiments. In the future, this could be achieved by providing a dynamic traffic model based on situational driving anger to predict changes in driving behavior as participants drive continuously in the lane.

In the future, data on mood changes can be collected using more objective emotion recognition, and thus left-turn behavior can be studied. Secondly, driver education training could also improve driver cognition and thus mitigate traffic accidents from the driver's perspective. Finally, driving devices could be improved using artificial intelligence technology, for example, by adding sensors that recognize gap encroachment so that drivers' left-turning behavior can be well monitored and prompted.

## Conclusions

This study aimed to investigate the effect of situational driving anger on left-turn driving behavior under different lighting conditions. The unique contribution of this study is to provide some causal relationships between illumination levels, situational driving anger, and left-turn driving behavior by improving the experimental design and strictly controlling for potential confounding factors. The findings indicated that light conditions significantly affected participants' anger intensity and that anger intensity was substantially higher at night than during the day. Secondly, light conditions were more responsive to male drivers' anger situations, different from previous studies. Thirdly, angry participants were more likely to drive dangerously when turning left at night, as the gap between receptive and post-encroachment time (PET) was shorter and more careless at night than during the day.

Based on the findings of this study, the following recommendations are made to reduce the adverse effects of situational driving anger. Firstly, develop real-time anger recognition technology and suggest necessary mitigation strategies to help drivers manage their emotions. Secondly, the negative attitude of drivers in an angry mood is an influential factor leading to accidents. Therefore, learning in driver education can improve the ability to identify and change negative driving attitudes correctly. Thirdly, hazard warning systems can be installed in vehicles to effectively help angry drivers access road information. Finally, light environment recognition sensors can be developed to enable drivers to recognize light better and be paired with appropriate intervention strategies.

## Data Availability

The data and material of the current study will be available from the corresponding author upon reasonable request.
